# Iconoclasm of normative structures: exploring queer ageing in “*Kaathal: The core*” by Jeo Baby

**DOI:** 10.3389/fsoc.2025.1557332

**Published:** 2025-07-03

**Authors:** A. C. Preethika Shree, G. K. Chithra

**Affiliations:** Vellore Institute of Technology (VIT), Chennai, India

**Keywords:** queer, ageing, male homosexuality, gerontology, iconoclastic

## Abstract

**Introduction:**

This research critically examined the portrayal of older gay men through an iconoclastic perspective in the Malayalam film *“Kaathal: The core,”* directed by Jeo Baby, focusing on the intersecting identities of age, marriage, and homosexuality as depicted in the film, further exploring how these overlapping facets shape the characters’ lives and experiences.

**Methodology:**

A qualitative research approach was employed to examine the representation of older gay men in the film. The study focused on narrative analysis of key scenes, dialogues, character portrayals, and cinematic elements to explore how the intersection of age, sexuality, and cultural norms is depicted.

**Results:**

The film narrative explored the challenging circumstances faced by the older gay characters, Mathew Devasy and Thankan, within a heteronormative cultural framework, particularly as of those who stay mute on their identity, remaining isolated and vulnerable. The research discloses the hetero character Omana’s display of significant agency, challenging traditional views on family and belonging.

**Discussion:**

The film critiques the rigid heteronormative framework of traditional family structures, offering alternative perspectives through non-traditional kinship and support systems. By addressing the intersection of age, sexuality, and cultural norms, this research emphasizes the importance of expanding queer representation to include diverse identities and life stages, paving the way for more inclusive cinematic discourses.

## 1 Introduction

Cinema has become a significant part of humanity and existence in popular culture. It portrays the differences in shifting public lifestyles, varying in social, political and cultural norms. It occupies and even alters the behavior, and belief systems of the common audience. Indian cinema, though existing for more than a century, is still hesitant, in the expression of queer people on screen. Though it was gradual, the evolution of portraying the community as a comical figure, or as a subject of ridicule has improved to sensitive narrations in the contemporary times demanding maturity and respect in the matter among the citizens. Despite the recent decriminalization of Section 377, by the Supreme Court, a British colonial penal code, that criminalized all sexual acts “against the order of nature,” there are still doors to be unlocked and milestones to be accomplished in the concerns of the community, regarding social, political, and legal. Cinema as a tool of artistic expression has been an influencing factor among general viewers both optimistically and at times, producing challenging realities, making screen space utilization on improving the quality of people’s lives equally important as commercial entertainment. Among the four major film industries of Indian cinema, Bollywood, Kollywood, Mollywood, and Tollywood, the least involvement in establishing queer visibility in mainstream movies is the Malayalam film industry. Ageism, like sexism and racism is as a systematic stereotyping of, and discrimination against people because they are older. I see ageism manifested in a wide range of phenomena, on both individual and institutional levels - stereotypes and myths, outright disdain and dislike, simple subtle avoidance of contact, and discriminatory practices in housing, employment, and services of all kinds (Qtd. in Butler 1989, p. 139). Ageism, as Judith Butler, an American feminist philosopher and gender studies scholar, observes, that the non-normative, vis-à-vis youth society is largely structured on the assumption that the majority is not older. But it is important to note that ageing is a universal process. While ageism can be linked to racism, sexism, or homophobia, unlike the latter, it affects everybody, regardless of class, caste, race, ethnicity, and gender or sexual identity. Therefore, the “aged” cannot be an exclusive identity or demographic category (Paromita and Kaustav, 2024). Ageing is shaped by diverse factors such as sexuality, gender identity, socio-economic status, cultural norms, etc (Wikipedia Contributors, 2025). These intersecting identities create varied ageing experiences, with some individuals facing heightened social and emotional challenges. Mainstream narratives, however, often depict ageing as a universal process, focusing on physical decline and life reflection while overlooking the unique struggles of marginalized groups. Older LGBTQ+ individuals may encounter isolation, discrimination, and unresolved identity conflicts, making their experiences distinct from conventional ageing narratives. This highlights the need for more inclusive portrayals that reflect the complexities of queer ageing.

Although representation of queer lives has gained visibility over time, accounts of elderly gay men remain underrepresented which makes it difficult to fully appreciate the subset of queer experience that is ageing. Existing not only in the Indian Diasporic imagination but also in American mainstream and regional narratives, older queer figures are often rendered invisible, or shown as elderly, single people without sexual/racial agency. It is argued that this neglect is not just a deficit but also a type of stereotyping that poor representation of older LGTBQ+ people is an understated premise and dominance in LGBT relations is assigned to youth only (Ferrer C., 2017; Ferrer J., 2017). In culturally conservative societies rooted in heteronormative ideas like India, older queer people must juggle between adhering to familial ties and accepting their sexual identity. Clearly there is a need to question the simple “adding in” of gay and lesbian identities as if they are “just another” cultural group (Hicks and Watson, 2003). Hence, the irony of older gay men being left out of the social and romantic scenes must be addressed. It is argued that a queer ageing approach would encounter older people not just as bodies with sexual needs, but also as erotic beings with diverse sexualities to be celebrated and desired (Hughes, 2006). They are frequently subjected to social isolation, hyper sexualization, or invisibility, causing considerable difficulty in social interactions or dating. Older queer individuals do not only share the experience of ageism, as seniors, Judith Butler noted that they face unique social discrimination over their sexual identity as well (Butler, 1980). Ageing may witness numerous changes for queer elderly; however, to be older and queer is another fraught identity, especially from regions characterized by conservative parameters. In India, a country that remains entrenched in social conservatism, the themes of queer elders are largely neglected, notwithstanding the country’s architectural situations that lend themselves to radical transgressive populations such as queer persons. Jeo Baby, the director of *“Kaathal: The core,”* offers scenes that allow viewers to appreciate multiple facets of older gay men, including their emotional and sociocultural environments, especially that of a sexually suppressed married man. This is potentially diametrically opposed to stereotypes in popular media that associate queerness with youth and rebellion. At the convergence of gerontology and queer theory, this paper looks at the question of how *“Kaathal: The core”* depicts the realities of senior homosexual men within their families and their conservative Kerala culture. It looks at how these characters can cope with their queer selves as they experience the process of ageing and abiding to social standards and expectations, to argue how Indian screen narratives might subvert stereotypes about queer confessors, thus allowing discussions of visibility, relational power and sexual freedom of older queers. Also, it considers how the value systems promoted by the family, placing heterosexual norms at their core, are detrimental to older homosexual men’s self-representation and expression in cinema, thereby forcing them into a silent or sterilized position. In this way, this study contributes to the current debate about the relationship between age and queerness and the emerging campaigns in the UK and the US promoting more intersectional approaches to LGBTQ narratives in Indian cinema. In India after the decriminalization of homosexuality, in 2018, the representation of queer themes in films and literature began to grow, but quite slowly” (Johar, 2018). The invisibility of elder queer figures leads not only to the silencing of their stories but also the false impression that queer life does not extend beyond the youth infusion hence, skipping the natural history of queer identity as a sum of (Browne and Nash, 2010). For most of them, senescence adds further dimensions of social disinvestment and family expectation to the complexities of queer identity, especially in cultures where heterosexuality and bearing children are considered essential to family and community inclusion (Suh and Son, 2020).

## 2 Review of literature

Queer aging, an integral aspect of queer studies, remains less explored in both academic, research and cultural representations, particularly in the context of male homosexuality. While contemporary discourses on queer identities have gradually gained visibility, the focus has predominantly rested on younger individuals, often aligning narratives with themes of self-discovery, coming-of-age, or romantic relationships. This skewed representation perpetuates the marginalization of older queer individuals, leaving their experiences, emotions, and identities largely invisible. The portrayal of ageing gay men in Indian cinema reflects this imbalance, where the narrative lens seldom shifts beyond youthful explorations of identity. In rare instances where older queer characters are depicted, they are frequently framed within stereotypes of isolation, deviance, or sexualization, effectively rendering their lived realities peripheral to the central narrative. This gap in representation and analysis overlooks the intersectionality of ageing and queerness, particularly the emotional and social complexities faced by older gay men in societies deeply entrenched in heteronormative and patriarchal structures (Bose, 2009). Conventional gerontological studies tend to emphasize a sex decline or absence of sexual desire or relational needs in older adults (Calasanti and Slevin, 2006). This is particularly the case for queer people, for whom the performance of desire, wanting, or having relationships can already be contentious. For older gay men in the Indian context, this dual invisibility, of being elderly and queer, exposes them to prospects of experiencing social and romantic lives, which leads to a kind of “double alienation” (Butler, 1980). The idea of mobility through space and time brings in coordination and structure to the sound lives of gay men, and age can be an aspect towards this (Pullen, 2012). Therefore, this study places *“Kaathal: The core”* in specificity to gerontological discourse and describes it as a cultural text that explores male homosexuality as an essential or a basic unit useful for analysis as opposed to being related. Their lives reflect the very quiet struggles and resilience of older queer individuals who have managed to “live quiet lives” and “discrete LGBT lives” taking care of family first (Hicks and Lee, 2006). Such characters also serve to challenge the norms of the Indian society that views elderly adults as not being sexual beings, with most of the gay stereotypes focusing on married couples, especially Indians. This analysis acknowledges that emphasis on families of older queer individuals in the media has been a strike against ageing invisibility that seeks to erase such constructs. Such analysis seeks to undermine the notion that *“Kaathal: The core”* is somehow a progressive film for Indian cinema, it discusses the much broader issues relating to intersectional inclusivity in India, particularly how the older population is integrated into the activism space and campaigns, societal perceptions, post legalization of gay marriage. Slowly, over the years western culture began to influence the media and decrease the viable hyper masculinity portrayal of queers, increasing visibility, and providing representation for more complex queer characters in media (Nicolazzo, 2021). However, such a visibility level has not been reciprocated to the older LGBTQ population (Sen, 2016). Violence against older LGBTQ people can be over-ridden by the sense of emotional belonging and relatedness to core supportive queer family units not based on conventional definitions of family, which the film explains. Unlike in Indian society however, avunculate attachments or more informal constellations known as the homosexual brotherhood as referred to by Loren P. Beth have emerged for older queer Indians as lifelines gained out of such oppression (Weston, 1991). *“Kaathal: The core”* thus prompts concerns relating to the negotiability and the creativity of a queer person’s neighborhood in a context where they are immersed in a more conventional family setting. Rather than focusing on coming out as the primary narrative when studying the lives of older gay men, this research elevates the longitudinal diversity of queer lives as projected by *“Kaathal: The core.”* It is vital to enhance the understanding of sexuality, identity, and psychocultural integration of a family unit by adding another dimension of expanding the horizon of LGBTQ+ representation to include older age queerness in Indian cinematic discourses. This way, older adolescents who are queer are not left out of the understanding and appreciation of queer culture as this allows for a deeper understanding of them, as “queer” known to be a more civil and well-mannered term and society’s view towards them (Chatterjee, 2020). The movie emerges as a critical text in addressing the underrepresentation of queer ageing in Indian cinema. The film ventures into themes of societal conformity, familial obligations, and the suppressed identities of older gay men, offering a lens through which to interrogate the interplay between aging and queer identity. Through the character of Mathew Devasy, *Kaathal: The core* explores the challenges of navigating a queer identity in the later stages of life within the confines of a traditional family structure dominated by heteronormative values. Drawing upon the theoretical frameworks of gerontology and queer studies, this study examines how the film situates aging gay men as complex, multi-dimensional individuals, contrasting the erasure and invisibility often observed in cinematic portrayals. Furthermore, it critiques the impact of societal expectations, internalized shame, and cultural taboos on the emotional, sexual, and social lives of older gay men. By delving into the intersections of age and queerness, the study underscores the urgent need for nuanced narratives that go beyond youth-centric depictions of queer experiences and contribute to a broader understanding of the diversity within LGBTQ+ communities. This analysis situates the film as a significant cultural text that challenges dominant ideologies and invites discourse on the experiences of older queer individuals, particularly in conservative socio-cultural contexts. The film bridges the themes of gerontology, queer ageing, and iconoclasm by presenting ageing not as a limitation but as a site of resilience and redefinition for queer individuals. While gerontology often frames older adults as desexualized or socially disengaged, *Kaathal: The core* resists this narrative, highlighting how older gay men carve spaces for emotional and sexual fulfillment within restrictive societal frameworks (Hicks and Lee, 2006). The film’s iconoclastic approach reshapes the cultural imagination of ageing and queerness, advocating for more inclusive narratives that celebrate diversity across all life stages.

## 3 Research gap

### 3.1 Underrepresentation of older queer narratives in Indian cinema

While queer representation has improved post-decriminalization of Section 377, narratives focusing on older LGBTQ+ individuals remain scarce. Indian cinema predominantly portrays queer identities through the lens of youth, often ignoring the experiences of ageing queer individuals. *Kaathal: The core* provides a rare exception, highlighting the lack of focus on older queer lives in cinematic storytelling.

### 3.2 Lack of intersectional analysis in queer ageing

Existing literature often studies queer identity and ageing in isolation, failing to address how intersecting factors like age, gender, sexuality, and cultural context compound the challenges faced by older LGBTQ+ individuals. The intersectional experiences of older queer men in conservative, heteronormative societies like Kerala have not been adequately explored.

### 3.3 Overlooked iconoclasm in queer representation

Few studies explore how films like *Kaathal: The core* challenge and disrupt traditional narratives surrounding ageing, queerness, and family structures. This lack of focus undermines the potential for broader discussions on the resilience and agency of older queer individuals in conservative settings.

Despite the growing presence of queer narratives in Indian cinema, older LGBTQ+ individuals remain significantly underrepresented, with most portrayals fixated on youthful experiences. This absence marginalizes the realities of queer ageing, reinforcing the misconception that queerness is exclusive to youth. By examining *Kaathal: The core*, this study addresses this gap, demonstrating how the film highlights the emotional, social, and familial struggles faced by ageing queer individuals, particularly within Kerala’s conservative setting. Furthermore, existing scholarship often isolates queer identity and ageing, overlooking the compounded challenges faced by individuals who navigate both.

## 4 Methods

The researchers employed qualitative research methodology, and narrative analysis was utilized to examine important scenes, character development, and dialogues that dealt with and focused on the representation and experiences of older queer individuals, thus addressing the research question of how the film *Kaathal: The core* depict the intersection of age, queerness, and cultural conservatism in the lives of older gay men, further dealing with what this representation reveal about the challenges of queer ageing in Indian society.

### 4.1 Enhanced methodological approach

The film was viewed multiple times to develop an in-depth understanding of its narrative structure, character development, and thematic elements. Film transcripts, including dialogues, were transcribed and reviewed to capture both explicit and implicit messages. Each segment was coded based on recurring themes such as “Queer Invisibility,” “Familial Conflict,” “Iconoclasm,” and “Emotional Isolation.” Codes were grouped under broader thematic categories such as “Intersectionality in Queer Ageing,” “Legal and Cultural Resistance,” and “Emotional Consequences of Silence.” Visual elements such as body language, facial expressions, camera angles, and mise-en-scène were analyzed to uncover deeper emotional and psychological dimensions. For instance, the use of dim lighting in certain scenes, the religious chants, and confined spaces of home and church was interpreted as a visual metaphor. By implementing this structured analytical process, the study ensured a systematic exploration of the film’s narrative complexities, visual language, and cultural commentary.

### 4.2 Materials

The primary data source for this study is the film *Kaathal: The core*, directed by Jeo Baby. The film has been analyzed in its entirety, focusing on key scenes, dialogue, and character interactions that highlight the experiences of ageing gay men within their social and familial contexts. Specific scenes that reveal tensions between the characters’ queer identity and traditional family structures has been analyzed closely, as they serve as focal points for understanding how intersectional identities manifest within the film’s narrative. In addition to the film, secondary sources including critical reviews, and existing scholarship on queer representation in Indian cinema are referenced to contextualize the findings and thus reinforce the analysis.

### 4.3 Procedure

Narrative analysis was employed as the primary qualitative method to explore the representation of older gay men in *Kaathal: The core*. The procedure for analyzing the film was adapted from established methods in cinematic narrative studies, where the film was viewed multiple times to identify key themes and data points relevant to the research question. Both visual elements, such as the characters’ body language and positioning within family settings, and verbal elements, including key dialogues, were carefully analyzed. The speech was transcribed, and subtitles were used to ensure precision in capturing the film’s implicit and explicit messages. This combination of visual and verbal data allowed for a comprehensive understanding of the nuanced representation of older gay men in the film, providing critical insights into their negotiation of identity, agency, and belonging within a conservative cultural framework.

The film engages with gerontological and queer theories by addressing themes of invisibility, repression, and resilience. The film reflects gerontological concerns highlighted by Hughes (2006) and de Vries et al. (2013), who emphasize the “double marginalization” faced by older LGBTQ+ individuals. Mathew’s concealed identity aligns with Westwood’s (2016) concept of the “silent generation,” where older queer individuals suppress their identities to survive in conservative societies. While reinforcing this invisibility, the film also challenges it by depicting Mathew’s eventual confession and Omana’s decision to divorce, reflecting Minichiello (2015) view that older queer individuals negotiate their identities through quiet acts of defiance. Drawing from Butler’s (1990) theory of performativity, the film illustrates how Mathew performs heterosexuality to meet societal expectations, while Sedgwick’s (1990) theory of the closet is reflected in Mathew’s prolonged silence and the emotional toll it exacts. By embedding these struggles within Kerala’s cultural framework, the film aligns with Crenshaw’s (1991) intersectionality theory, showing how age, sexuality, and cultural pressures compound marginalization (Sharma and Samanta, 2020). Ultimately, the film blends elements of repression, visibility, and resilience, offering a layered critique of queer ageing within a regional Indian context.

## 5 Findings

**Figure 1:**
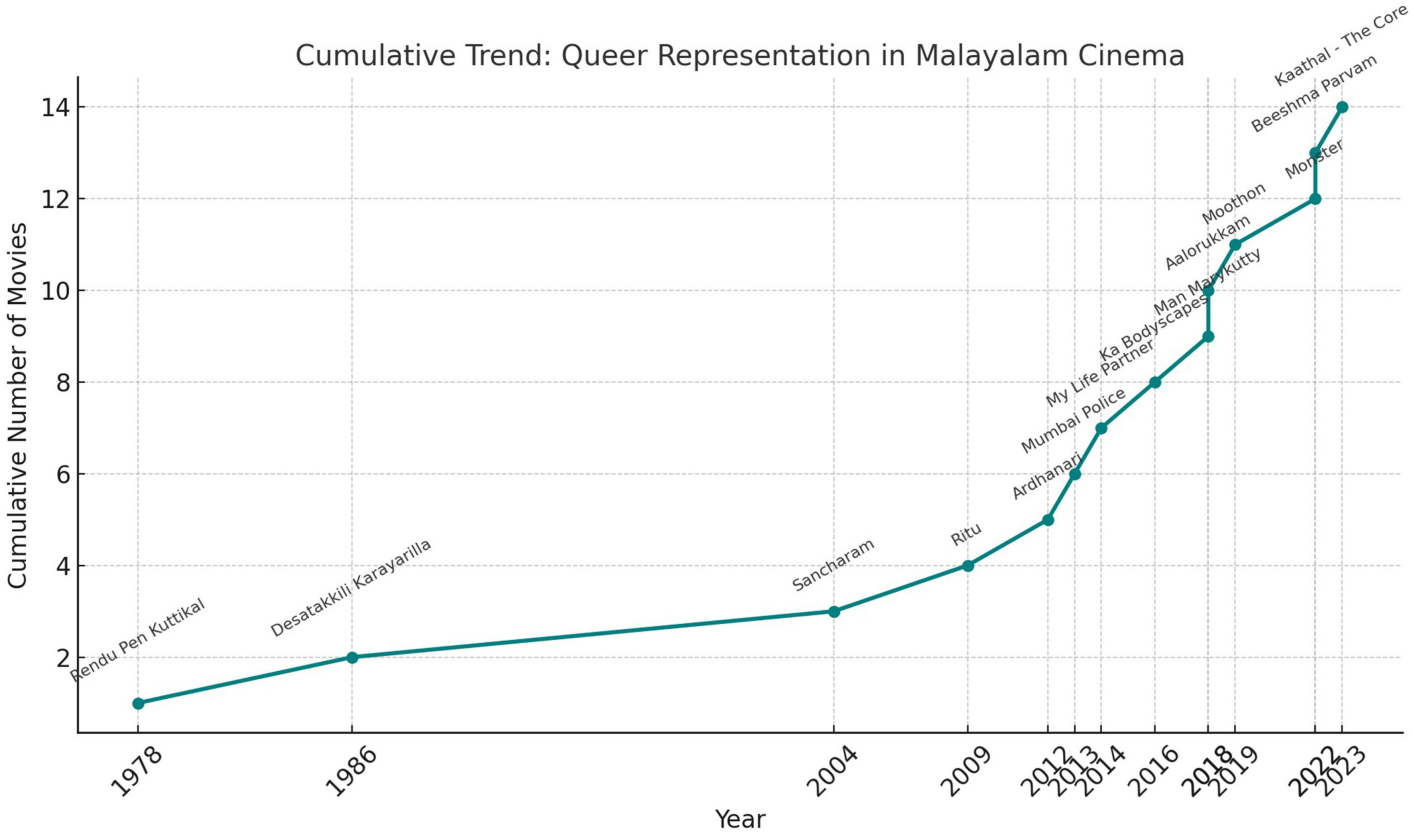
Queer representation in Malayalam cinema.

The research reveals the evolution of queer representation in Malayalam cinema, highlighting both progress and persistent challenges. The analysis of *Kaathal: The core* alongside an examination of LGBTQ+ portrayals in Malayalam cinema, demonstrates the growing willingness of filmmakers to address the complexities of queer lives, albeit unevenly across decades. The findings in Figure 1, show that early portrayals, such as in Rendu Penkuttikal (1978) and Desatakkili Karayarilla (1986), were subtle and coded, reflecting the cultural and societal limitations of their time. These films cautiously hinted at queer themes without overt acknowledgment, allowing them to exist within conservative cinematic spaces while opening the door for future narratives. The shift in the 2000s, marked by films like Sancharam (2004) and Ritu (2009), presented more explicit queer stories, though they often fell into tragic tropes. Sancharam, with its positive depiction of a sapphic relationship, offered an affirming narrative, while Ritu and others of the period reflected societal stigma, often ending in heartbreak or punishment for queer characters. In the 2010s, a broader and more diverse range of narratives began to emerge. Films like Mumbai Police (2013) explored the intersection of queer identity and professional life, while Njan Marykutty (2018) and Aalorukkam (2018) brought attention to transgender lives, showcasing resilience against societal and familial rejection (The Indian Express, 2018). However, even during this period, films like My Life Partner (2014) and Ka Bodyscapes (2016) illustrated the ongoing struggle against stereotypes and the censorship of queer content in regional cinema. The 2020s signify a notable increase in queer representation, both in volume and diversity (India Today, 2019). Films like Moothon (2019), Monster (2022), and Beeshma Parvam (2022) reflect attempts to integrate LGBTQ+ themes into mainstream Malayalam cinema, though not all succeed in breaking away from harmful stereotypes. *Kaathal: The core* (2023) stands out for its bolder exploration of queer ageing, a largely neglected topic. By focusing on older gay men and their struggles within a conservative societal framework, the film addresses the compounded marginalization caused by age, sexuality, and cultural expectations.

This analysis underscores the evolution of queer representation in Malayalam cinema, revealing that while progress has been made in bringing LGBTQ+ lives into the spotlight, significant gaps remain. Tragic endings, stereotypical depictions, and an overreliance on implicit portrayals highlight the need for more nuanced and intersectional narratives (Financial Express, 2018). The research further emphasizes the importance of exploring overlooked topics, such as queer ageing and the lived experiences of individuals navigating non-heteronormative identities in conservative societies. Films like *Kaathal: The core* represent a critical step forward, advocating for inclusivity and visibility in cinematic storytelling.

## 6 Analysis

The choice of narrative analysis in this study is particularly justified due to its ability to examine the intricate emotional, social, and cultural layers present in *Kaathal: The Core*. Unlike other qualitative methods such as thematic analysis or ethnography, narrative analysis is uniquely suited to dissect the film’s complex storytelling structure, character development, and visual language. Moreover, narrative analysis aligns with the study’s intersectional approach, as it enables the identification of layered identities and social hierarchies embedded in the film’s storytelling. Further, the film’s focus on the nuanced experiences of older gay men navigating familial expectations, social stigma, and internalized shame, makes narrative analysis particularly effective, allowing for a deeper exploration of how these themes are constructed and communicated.

### 6.1 Enhanced interpretive depth through cinematic techniques

By integrating cinematography, lighting, and body language into the thematic analysis, this study uncovers deeper layers of meaning that reinforce the emotional struggles faced by older queer individuals.

#### 6.1.1 Cinematography and spatial framing

The film frequently utilizes tight framing and close-up shots to capture Mathew’s internalized tension and emotional confinement. For instance, during Mathew’s moments of introspection, the camera focuses closely on his face, emphasizing his anxiety and the burden of living a concealed identity. These visual choices mirror the suffocating effect of social expectations and reinforce the theme of emotional isolation. In the scenes featuring Mathew and Thankan, the camera employs over-the-shoulder shots to frame their unspoken conversations. This technique places viewers in a voyeuristic position, symbolizing the pervasive social surveillance that controls their identities and actions.

#### 6.1.2 Lighting and symbolic contrast

The film’s deliberate use of low lighting and shadows emphasizes the tension between concealment and exposure. For example, the climax scene where Mathew confesses his apology to Omana and breaking down to his father before, unfolds in dim lighting, with shadows partially obscuring his face. This visual metaphor reflects the emotional weight of his hidden identity and underscores the theme of shame and secrecy. In contrast, brighter lighting is reserved for moments of social conformity—such as Mathew’s political campaign scenes—symbolizing the pressure to maintain a respectable public image. This contrast between dark and illuminated spaces reinforces the protagonist’s inner conflict.

#### 6.1.3 Body language and emotional suppression

Throughout the film, Mathew’s restrained gestures and averted eye contact highlight his discomfort and fear of exposure. His rigid posture during interactions with Thankan reflects the internalized shame and the anxiety of being scrutinized. In key scenes, such as Mathew’s political address or Omana’s confrontation, his stiff body language contrasts with Omana’s expressive movements, symbolizing her growing agency as she challenges patriarchal norms.

#### 6.1.4 Sound design and emotional undercurrents

The film’s restrained use of background music emphasizes moments of silence, creating an atmosphere of tension and emotional vulnerability. For example, in the rain-soaked car scene where Thankan reflects on his unspoken love, the absence of dialogue paired with ambient sounds amplifies his emotional turmoil, reinforcing the theme of love lost to societal constraints.

### 6.2 Iconoclasm and the politics of marriage, sexuality, and identity

According to Butler, the notion of gender is a construct of the society, where people (especially the queer communities) struggle to meet the set standards of gender roles, fearing getting pushed out from the mainstream. Prajoth and Priya (2024). They are in a way, strained to become or perform as another person to overcome the title of being an outcast, other type, inferior, etc. Tacium (1993). A misconception about sexuality has been built that “heterosexuality is natural.” This cultural construct, utterly excludes queer identities, be it, artistic, literary or political. As Eve Sedwick explains in the “Epistemology of the closet,” when two elements of conflict always coexist, which are not of equal powers, one oppresses the other, whereas queer identity politics challenges this, opposing the ideas imposed and the social construct. This power play demands an oppressor and an oppressed. The sympathy shown by the hetero public towards the queer minority, is a prevailing problem (Warner, 1999). Postmodern narratives have taught us to fight conflicts and grand narratives, thus paving way for equalizing this powerplay, eliminating the need to express superiority over the other. This sympathy trend is visible in many contemporary films. Amidst such narrations, offering fresh perspective on the lived experiences of older gay men is *Kaathal: The core* directed by Jeo Baby starring Mammootty as Mathew Devasy and Jyothika as Omana Philip Mathew. The plot develops as Mammootty embarks on a political journey as left-wing independent candidate in the election. The film subtly raises questions on what it means to grow older as a queer person in a culture where queerness remains a taboo, particularly in the context of a late-life companionship, loneliness, and societal erasure of queer identities. The film establishes subtle yet powerful undercurrents that reflect the experiences of older queer individuals, especially of those who oblige to societal standards of heteronormativity. Coming out of the closet is a traumatic experience in the life of gay individuals as the fear of ostracization may at times force them to try to behave “normal.” In this pursuit, most of them face extreme identity crisis as they find themselves enmeshed in the quagmire of meaningless marriage associations, emotional havoc, mental instability, suicidal thoughts and ultimately the unrepresentable pain of negotiating oneself, beyond every other suffering. Prajoth and Priya (2024). The courtroom scene in *Kaathal: The core* serves as a profound commentary on iconoclasm, particularly through the character of Omana’s lawyer Ameera, who challenges the entrenched societal norms surrounding marriage, sexuality, and gender norms. The lawyer’s argument directly critiques the longstanding cultural ideologies that have kept LGBTQ+ individuals, and their heterosexual spouses bound within the same oppressive framework, rendering them powerless to challenge the status quo. By calling attention to the pervasive silence forced upon women and marginalized communities, the scene subtly dismantles these traditional structures, aligning with an iconoclastic approach to social norms and legal systems.

Lawyer Ameera. The women of our society still do not have the luxury of choice, to come out of their families and take their own stand (Baby, 2023, 01:11:59).

Iconoclasm, traditionally seen as the act of destroying or challenging established traditions and values, takes on a unique form in this narrative. The lawyer’s reference to the decriminalization of Section 377 serves as a symbolic act of defiance against not just the law itself but also the centuries-older patriarchal ideologies that have long governed Indian society. The idea that Omana must wait for the legal system to change before she can take control of her life speaks about the power dynamics, in the place. It’s an ironic reflection on how, to assert personal freedom, individuals must often first seek legal validation from systems that have historically oppressed them.

Lawyer Ameera. And, if you ask why she took this long, let me remind you it was only last year that Section 377 was decriminalized. Suppose Omana had gone ahead with such a case before that as per then existing law, Mathew would have considered engaging in a criminal offence. If not anything, she must have been aware that her husband is not a criminal. And this lady had to wait until 2018. Just because we had such a law in our country (Baby, 2023, 01:12:13).

The lawyer’s quoting of Justice Malhotra’s words while passing the decriminalization verdict, “History owes an apology to the members of this community” (Baby, 2023, 01:12:40), are not only a critique of the legal system but a challenge to the social norms that have long marginalized both queer individuals and their spouses. Furthermore, the lawyer’s invocation of the statistic about the high number of queer individuals married to straight partners, “more than 80% of homosexuals are married to a straight partner,” highlights the societal pressure to conform the heteronormative ideals. This powerful statistic becomes a critique of the institution of marriage itself, showing how deeply ingrained cultural norms have forced queer individuals to hide their identities and live in relationships that are often emotionally suffocating. The film, through this scene, critiques the very idea of marriage and challenges the role it plays in maintaining heteronormative standards. Omana’s decision to divorce, after years of enduring this oppressive silence, is nothing short of iconoclastic. It represents a breaking away from traditional marital expectations and a call for societal transformation, where personal freedom and authenticity are not constrained by legal or cultural boundaries. The scene ultimately contributes to a larger conversation about how breaking societal norms, particularly those related to marriage and gender, can be seen as an act of resistance. In this context, Omana’s divorce is not just an emotional decision, but an iconoclastic stand against a society that continues to enforce conformity, at the cost of hiding the fact. By making this decision, Omana is not only reclaiming her own life but is also calling attention to the oppressive structures that govern the lives of both LGBTQ+ individuals and those, they are entwined with. Thus, this moment in the film transcends the personal, serving as a critique of larger societal and cultural systems, urging a rethinking of traditional views on family, marriage, and identity.

### 6.3 The burden of silence: cost of authenticity in queer lives

The experiences of older gay men in conservative societies like Kerala reveal how deeply entrenched societal norms weaponize shame and ridicule to enforce conformity and silence. In *Kaathal: The core*, these dynamics are poignantly portrayed through characters like Mathew and Thankan, whose emotional, sexual, and social lives are shaped by a culture that refuses to acknowledge or validate their identities. The politics of shame becomes a powerful tool to marginalize older queer individuals, rendering them invisible in public spaces while deeply isolating them in private. The scene where Thankan and his nephew Kuttayi are mocked by random men on the street, “Hey, Thankan. Let the boy ride the bike. And you sit at the back. That will be convenient for you” (Baby, 2023, 00:39:18) is a stark depiction of how societal ridicule eroding the dignity and authenticity of queer individuals in public spaces. The casual tone of the taunt reflects the ingrained nature of societal prejudice, where non-conformity to heteronormative ideals is seen as a subject of humor and derision. It exemplifies how public spaces are often weaponized against queer individuals. For older gay men like Thankan, this ridicule is not just an insult but a reminder of their societal erasure. It underscores the collective discomfort with their visibility, particularly because they challenge heteronormative narratives that equate age with sexualization and conformity to traditional family roles. Thankan’s silent response to the mockery reflects the emotional toll of enduring such humiliation, where dignity is sacrificed for survival in a judgmental world. This scene is a microcosm of the daily humiliation endured by queer individuals like Thankan, who must carry the burden of silence to protect themselves from overt marginalization and violence. Mathew’s reluctance to separate from Omana is not driven solely by love or partnership but by the societal pressures that bind him to his role as a husband, father, and public figure. In a deeply patriarchal and heteronormative society like Kerala, marriage is often seen as a sacred institution that validates a man’s masculinity, respectability, and place within the family unit. For Mathew, stepping away from this institution would mean not just personal loss but also societal scrutiny, shame, and the erosion of his carefully constructed identity as a family man and political candidate. This fear of societal judgment is so pervasive that it compels him to sacrifice his true self in favor of a life marked by compromise and concealment. This is evident in the scene, Mathew. I… do not want to get separated from Omana” (Baby, 2023, 00:44:40). The scene poignantly captures how societal norms can imprison individuals, pushing them to sustain relationships that ultimately deny them the dignity of living as their true selves. It reveals how societal expectations force queer individuals to prioritize appearances over authenticity. For Mathew, the fear of losing familial and social acceptance outweighs his desire to live truthfully. The internalized shame of being unable to reconcile his public and private selves leads to emotional fragmentation, where his sexual and emotional needs are repressed to maintain societal validation. Shame also acts as a barrier to intimacy and self-expression, particularly for older gay men navigating their sexual lives. In societies that stigmatize queerness, expressions of love and desire are often ridiculed or deemed inappropriate, especially for individuals who do not conform to conventional notions of youth and attractiveness. Thankan’s grief, captured in the rain-soaked car scene, reflects not only the pain of unspoken love but also the societal rejection that has forced him to suppress his desires. The inability to express or act on these emotions without fear of ridicule or rejection highlights the heavy cost of shame on the sexual lives of older queer individuals. By intertwining the struggles of Mathew, Thankan, and Omana, the film critiques the oppressive structures of heteronormativity and patriarchy, exposing the emotional toll of conformity and inauthenticity.

### 6.4 Morality, marginalization, and resistance: the role of church and community in *Kaathal: The core*

The intersection of the church, community, and societal structures plays a central role in *Kaathal: The core*, revealing how deeply entrenched cultural and religious values govern personal and public lives, especially for queer individuals and their families. The film portrays the church and the community not as spaces of support or compassion but as institutions that weaponize morality, shame, and conformity to marginalize those who deviate from heteronormative norms. Omana’s brother’s reaction to her decision to file for divorce encapsulates the community’s patriarchal and moralistic stance on marriage. His statement, “Such trivial issues are normal in family life” (Baby, 2023, 00:25:53), dismisses Omana’s grievances as insignificant, undermining her autonomy and right to seek a fulfilling life. The suggestion that divorces at her age would scandalize the family, reveals how community reputation, rather than individual wellbeing, is prioritized. By framing Omana’s actions as a threat to her daughter Femi’s marital prospects, the brother uses shame as a tool to silence her, reinforcing the patriarchal control exerted by both family and community. The role of the church is equally complicit in perpetuating stigma and exclusion. The scene of the prayer, “O virgin of virgins, O merciful mother. Despite not my petitions, but in thy mercy hear and answer me” (Baby, 2023, 00:28:23) highlights the performative religiosity embedded in the community. This prayer, invoking mercy and compassion, is starkly contrasted by the church committee’s decision to expel Mathew from the parish, “Whatever it is, among the believers in the parish, it has become a talk” (Baby, 2023, 00:57:30) Instead of offering understanding or acceptance, the church becomes a moral authority that ostracizes individuals like Mathew, enforcing rigid notions of purity and propriety that leave no space for queer identities. The political community, too, plays a significant role in reinforcing societal stigma. When Mathew’s homosexuality becomes public knowledge, his party members express concern over its potential impact on the party’s image, “No one from a pious Christian family will vote for us.”

Despite their proclaimed ideology of respecting individual integrity, “Our ideology is such that we respect the integrity and identity of an individual (Baby, 2023, 00:32:44). This acceptance remains superficial, as it is quickly overridden by the fear of losing votes and public support. The opposite party’s campaign against Mathew amplifies this prejudice, framing his candidacy as a moral threat, “What influence will this create on our kids? Can you imagine the situation of our land if he becomes the Panchayath member?” (Baby, 2023, 00:35:35). These reactions reveal the political community’s complicity in perpetuating societal homophobia, using morality as a weapon to delegitimize queer individuals in public spaces. The film also depicts how ridicule and public humiliation are employed to enforce conformity. The men mocking Thankan in the streets, “Let the boy ride the bike, and you sit at the back. That will be convenient for you” (Baby, 2023, 00:39:18) reflect the community’s casual cruelty towards those who deviate from traditional gender and sexual norms. This ridicule not only isolates Thankan but also reinforces his invisibility within the larger social structure. Similarly, Mathew’s lawyer highlights the lack of legal frameworks to address such cases in Kerala, “There are not many cases or laws for us to refer to” (Baby, 2023, 00:42:14), revealing the systemic neglect and marginalization of queer individuals in both legal and social institutions. Omana’s lawyer sheds further light on the societal pressures that force queer individuals into heterosexual marriages, “Many homosexuals in their marriage life, because of their partner’s compulsion, or to pretend themselves as straight, have indulged in sex like this” (Baby, 2023, 01:08:10). This statement underscores the role of the community and family in coercing queer individuals into silence and conformity, often at great emotional and psychological cost. The lawyer also remarks on monogamous homosexual relationships, “There are people who maintain monogamous homosexual relationships for a long time,” highlighting how such relationships, though genuine, remain invisible and stigmatized in conservative societies. Through these scenes and dialogues, *Kaathal: The core* critiques the role of the church and community in perpetuating stigma, shame, and exclusion. These institutions, rather than offering acceptance or understanding, become enforcers of societal conformity, policing morality and punishing those who deviate from their norms. The film powerfully exposes the cost of this ostracization, showing how it fractures relationships, isolates individuals, and forces them to live inauthentic lives. At the same time, it challenges these structures by portraying the quiet resilience and humanity of its queer characters, urging viewers to question the oppressive systems that dictate personal and collective lives.

### 6.5 The weight of unspoken truths: love, loss, and the emotional toll of silence in *Kaathal: The core*

In *Kaathal: The core*, the characters of Mathew and Thankan embody the deep emotional toll of silence, exploring how unspoken truths fracture relationships, suppress identities, and leave lives haunted by regret. The monologues of both characters—Mathew addressing his father and Thankan reflecting on love—converge thematically to illustrate the weight of fear, societal expectations, and unfulfilled love in queer lives. Mathew’s confrontation with his father is a moment of raw emotional reckoning. In his monologue,

Mathew. You raised me, giving me all freedom. You understood all my needs. But when it came to this, you could not understand me.

He lays bare the pain of being misunderstood by the person he trusted most. This scene captures the intergenerational gap in understanding and acceptance of queer identities. His father’s advice in the past that everything will be alright if Mathew got married, reflects the societal norm of using marriage as a cure-all for queerness, a means to enforce conformity rather than address the truth of Mathew’s identity. The tragic irony in Mathew’s question, “Is everything alright now?” underscores the irreversible damage caused by such well-intentioned but misguided actions. His words reflect not only his personal suffering but also the collateral damage inflicted on Omana, whose life was equally disrupted by this concealment. This scene illuminates the devastating cost of silence and the societal obsession with appearances. Mathew’s life, lived as a “drama” to appease societal and familial expectations, becomes a metaphor for the struggle faced by countless queer individuals forced to prioritize external validation over internal truth. The toll of this suppression manifests in guilt and regret, as Mathew blames himself for ruining not just his life but also Omana’s, a woman who became an unwitting participant in his concealment. This moment is a poignant reminder of how silence, while protecting individuals from societal backlash, creates emotional wounds that ripple outward, affecting everyone involved. Similarly, Thankan’s reflection,

Thankan. “Everyone fears losing the ones they love. But there are people who miss out on love because of that fear.” captures the heartbreak of love lost to silence and fear. His words resonate with the quiet despair of living in a world where expressing his true feelings would invite ridicule, rejection, and ostracism. The pain of unspoken love, compounded by the societal stigma of being an older gay man, reflects the unique vulnerability faced by queer individuals in conservative societies. Thankan’s grief is not just about losing a chance at love with Mathew but also about the broader impossibility of living authentically in a society that denies him his dignity. Both Mathew and Thankan’s narratives converge on the theme of silence as a double-edged sword. On one hand, silence protects them from immediate judgment and harm; on the other, it isolates them emotionally and traps them in lives of unfulfilled potential. Mathew’s inability to speak his truth and Thankan’s fear of openly loving both stem from the same societal pressures that weaponize shame and conformity against queer individuals. Ultimately, *Kaathal: The core* presents a haunting commentary on the cost of silence in queer lives. Mathew’s and Thankan’s stories are a testament to the emotional burden of living inauthentically, driven by fear and societal expectations. The film challenges viewers to confront the structures that perpetuate this silence and to imagine a world where love and truth are not constrained by fear. By giving voice to these unspoken truths, *Kaathal: The core* stands as a powerful call for empathy, understanding, and the dismantling of oppressive norms that continue to marginalize queer lives.

## 7 Discussion

### 7.1 Gerontology and queer ageing

Gerontology, the study of ageing, has traditionally focused on the physiological and social implications of growing older. However, for queer individuals, especially older gay men, ageing introduces unique challenges. Calasanti and Slevin (2006) emphasize that traditional gerontological studies often neglect the relational needs and sexual desires of older adults, especially in the context of non-heteronormative sexual identities. For older gay men, this age-based invisibility is compounded by cultural conservatism. Butler (1980) explores the concept of “double alienation,” where older gay individuals face both ageism and homophobia, making them particularly vulnerable in family-centric societies like India. In this context, the invisibility of older gay men in Indian cinema reflects broader societal tendencies to marginalize non-heteronormative sexual identities, exacerbating their social and emotional isolation. Gerontological frameworks offer critical insights into the intersection of ageing and queer identities. Ageism, as defined by Butler (1989), reinforces the invisibility of older individuals, particularly within the LGBTQ+ community, where cultural narratives often prioritize youth and vitality. Older queer individuals experience “double alienation,” grappling with the compounded effects of ageism and homophobia (Butler, 1980). Studies highlight that older LGBTQ+ individuals are frequently desexualized and marginalized, their identities reduced to stereotypes that deny their emotional and sexual agency (Calasanti and Slevin, 2006; Suh and Son, 2020). In *Kaathal: The core*, the lives of Mathew and Thankan reveal how conservative cultural values in Kerala exacerbate this marginalization, limiting their opportunities for self-expression and social belonging.

### 7.2 Queer ageing and cinematic representations

**Figure 2:**
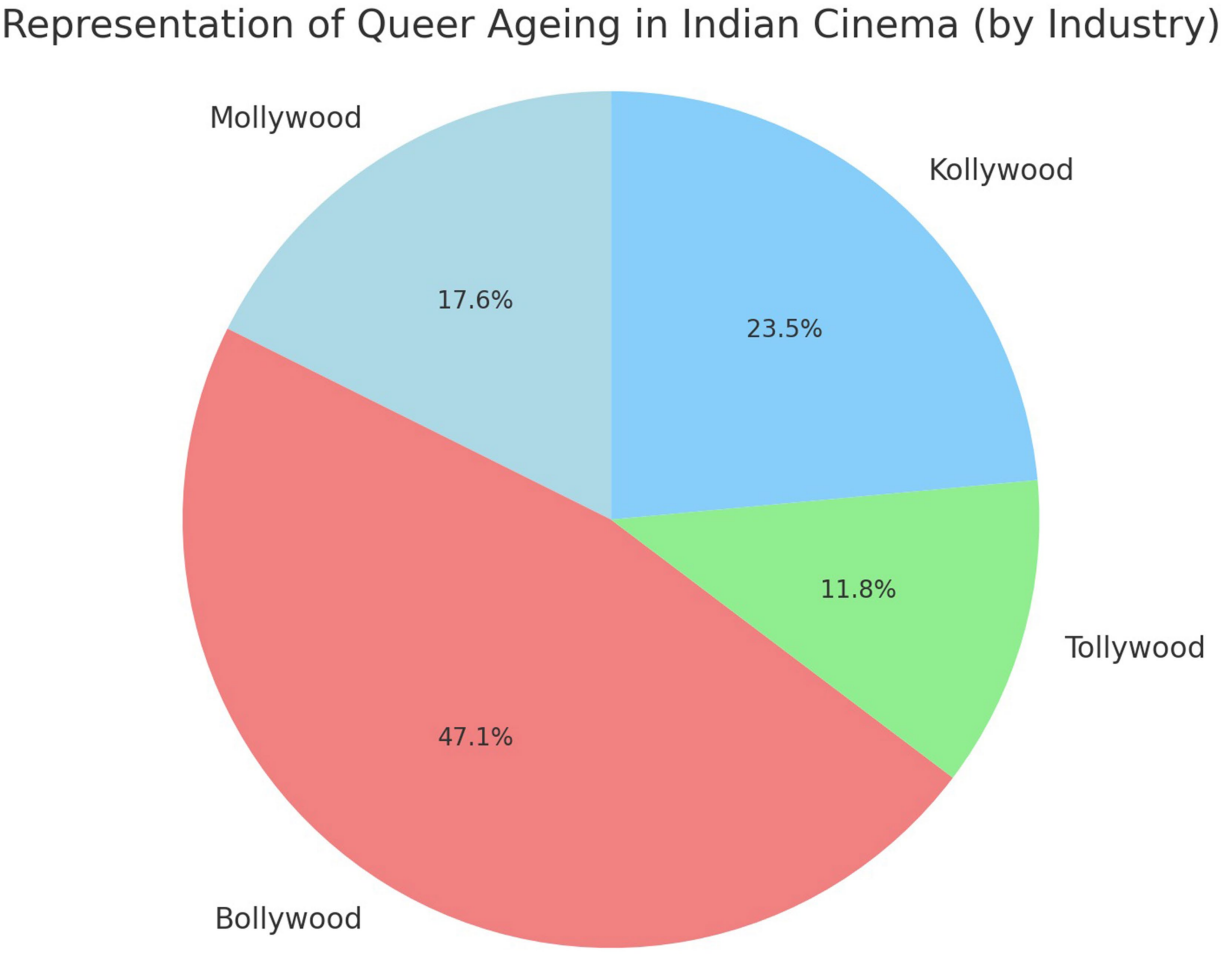
Representation of queer ageing in Indian cinema.

Ferrer C. (2017) and Ferrer J. (2017) critiques the absence of older LGBTQ+ figures in mainstream media, asserting that queer narratives often prioritize youth, marginalizing the natural progression of queer identity into older adulthood. This is especially evident in Indian cinema, where queer characters are frequently associated with youth, rebellion, and a lack of familial ties. However, *Kaathal: The core* provides a rare representation of older gay men, showing them navigating familial expectations, societal pressure, and emotional isolation while also asserting their sexuality. This aligns with the work of Hicks and Lee (2006), who discuss the “closeted” nature of many older LGBTQ+ individuals, who are forced to live discreet lives due to societal rejection. The data in the Figure 2 chart highlights Bollywood’s dominance and the limited visibility of older queer identities in regional cinema., Malayalam cinema has also historically been hesitant to depict LGBTQ+ narratives, particularly those involving older individuals, and as evident in the chart contributes 17.6% representation underscoring the limited visibility of older queer identities, thus making *Kaathal: The core* a significant intervention. Cinematic portrayals have the power to influence societal attitudes by challenging entrenched stereotypes. By depicting older queer individuals as complex, multi-dimensional people rather than tragic figures or objects of pity, films can foster empathy, dismantle stigma, and normalize diverse identities. Positive portrayals, meanwhile, can inspire intergenerational dialogue, encouraging younger audiences to recognize the social struggles faced by older queer individuals and promoting acceptance across generations. The film explores the compounded marginalization faced by older queer individuals, who experience both ageism and homophobia within a patriarchal and family-centered society in Kerala’s conservative social framework.

The film chosen is uniquely suitable for examining queer ageing in the Malayalam context because it:

- Engages directly with Kerala’s cultural conservatism.
- Depicts older queer characters navigating emotional suppression and societal rejection.
- Uses iconoclastic storytelling to challenge heteronormative family ideals.
- Introduces a nuanced portrayal of queer ageing, an area largely overlooked in Indian cinema.

The film offers a distinctive portrayal of queer ageing that diverges from the youth-centric narratives commonly seen in Indian cinema, such as *Kapoor and Sons* (2016) (Batra, 2016) and *Margarita with a Straw* (2014) (Bose, 2014). While these films focus on younger characters exploring identity, *Kaathal: The core* addresses the emotional repression, familial obligations, and social stigma faced by older queer individuals. Comparatively, the film aligns with international works like *Love is Strange* (2014) (Sachs, 2014) and *Supernova* (2020) (MacQueen, 2020), which explore queer ageing, yet it uniquely embeds these themes within Kerala’s conservative sociocultural framework. Additionally, *Kaathal: The core* extends the discourse seen in *Aligarh* (2015) (Mehta, 2015) by emphasizing the long-term emotional toll of a closeted life.

### 7.3 Intersectionality in queer ageing

Queer ageing remains a marginalized area within the broader spectrum of LGBTQ+ studies, a reflection of the societal inclination to frame queer experiences primarily through the lens of youth and self-discovery. This tendency to focus on the younger demographic sidelines the nuanced realities of older queer individuals, whose experiences intersect with age, societal expectations, and personal struggles. The narratives of older gay men, in particular, are often marked by invisibility or relegation to supporting roles in dominant discourses, further perpetuating their erasure. While progress has been made in representing diverse queer identities in global media, Indian cinema has been slow to embrace these changes, particularly when it comes to exploring queerness in its multifaceted forms. Stories that center around queer ageing are rare, and those that do exist often rely on reductive stereotypes or cast older queer characters as symbols of tragedy or deviance. The absence of nuanced narratives leaves a critical gap in understanding how ageing impacts queer identities, particularly in patriarchal societies where heteronormative family structures dominate.

The concept of intersectionality is crucial in understanding the complex realities of queer ageing. Older queer individuals face layered forms of marginalization that intersect across age, sexuality, and cultural norms. In *Kaathal: The core*, this intersectionality is evident as the protagonist navigates the dual burden of being an ageing individual in a conservative society while grappling with his concealed queer identity.

### 7.4 Iconoclasm and breaking of norms

Iconoclasm refers to the act of challenging and rejecting established norms, beliefs, or institutions. In the context of *Kaathal: The core*, the film’s depiction of older gay men challenges traditional views of ageing, queerness, and family. As observed by Anupam and Tannishtha (2020), the marginalization of older LGBTQ+ individuals disrupts societal assumptions of sexual functionality and moral duty. The protagonists of the film, by asserting their identities despite familial pressures, represent a form of iconoclasm that critiques societal expectations about ageing and sexuality. Their stories reflect the quiet, often unseen resilience of older gay men, who continue to navigate societal and familial structures while carving out spaces for themselves within conservative environments. This iconoclastic portrayal of queer ageing resonates with the broader need for more inclusive representations of LGBTQ+ lives at all stages of life (Dyer, 1993).

### 7.5 Intersecting marginalizations: age and queer identity

#### 7.5.1 Queerness, ageing, and societal erasure

*Kaathal: The core* emerges as a groundbreaking cinematic exploration of the intersectionality between queerness and ageing, offering a poignant critique of the emotional toll imposed by conservative socio-cultural frameworks. By employing a narrative, intersectional, and gerontological lens, this research illuminates how the film critiques patriarchal and heteronormative systems while shedding light on the compounded struggles faced by older gay men navigating dual marginalization. At its core, the film delves into the nuanced realities of queer ageing. Gerontology provides a critical framework to understand how ageing amplifies the marginalization of LGBTQ+ individuals. As society emphasizes youth in both heteronormative and queer spaces, older individuals like Mathew are rendered invisible, their lived experiences erased from dominant narratives. *Kaathal: The core* confronts this erasure, challenging the societal obsession with youthful vitality and heteronormative ideals that disregard the dignity and authenticity of queer lives in later years.

#### 7.5.2 The burdens of conformity and a call for cultural transformation

Mathew’s story encapsulates the intersectional struggle of age and homosexuality, where his identity as an older gay man is stifled by societal expectations. The film portrays how patriarchal norms, compounded by ageist and heteronormative biases, demand conformity to roles of husband, father, and community figurehead. Mathew’s silence becomes both a shield and a prison, enabling him to navigate societal expectations while suppressing his authentic self. His inability to break free from these roles results in profound guilt, regret, and emotional isolation—burdens that deepen with age. The intersection of queerness and ageing exposes the inadequacies of cultural frameworks that fail to account for diverse identities and lived experiences, especially in older age. Gerontology, as applied in the film, reveals how ageing intersects with queerness to exacerbate social invisibility and emotional isolation. For Mathew, the cultural and familial constructs that dictate his role as a patriarch leave no space for his authentic self, forcing him to perform heteronormativity at great personal cost. This performance not only fractures his relationship with himself but also inflicts collateral damage on Omana, his wife, who is similarly trapped within patriarchal expectations. The iconoclastic nature of *Kaathal: The core* emerges through its bold challenge to patriarchal and heteronormative structures. By portraying older queer characters like Mathew and Thankan, the film disrupts dominant cultural narratives that prioritize youth and heterosexuality. It questions the legitimacy of societal frameworks that enforce rigid roles and appearances, advocating instead for a reimagining of familial, religious, and cultural systems. These systems, as critiqued in the film, perpetuate the marginalization of individuals who defy normative expectations, particularly those at the intersection of queerness and ageing. In addition to its critique of societal norms, the film also highlights the resilience of older LGBTQ+ individuals. It situates their struggles within a broader gerontological discourse, underscoring the importance of addressing the unique challenges faced by this demographic. *Kaathal: The core* transcends its narrative to become a powerful iconoclastic critique of societal structures that silence and marginalize queer lives, particularly those of older individuals. Its nuanced portrayal of queer struggles, combined with its bold critique of patriarchal and heteronormative frameworks, calls for a cultural shift toward inclusivity, empathy, and visibility. Through its layered storytelling and iconoclastic stance, *Kaathal: The core* invites society to dismantle its biases and embrace the authenticity, dignity, and humanity of all individuals, regardless of age, gender, or sexuality.

### 7.6 Interrelation of themes

#### 7.6.1 Queerness and ageing in Indian cinema

In Indian cinema, where family values are often glorified and heterosexual relationships are positioned as the societal norm, older queer characters face dual marginalization: as members of a queer community coping with the stigma and ageing individuals in a youth-oriented culture too. The Malayalam film *“Kaathal: The core,”* directed by Jeo Baby, is a bolder intervention in this context, presenting a layered narrative that interrogates the intersections of queerness and ageing. Through its central character, Mathew Devasy, the film explores the complexities of living as an older gay man in a conservative society. Mathew’s character challenges the traditional depictions of ageing men as either devoid of sexuality or confined to heteronormative roles, offering instead a poignant look at the hidden struggles of older queer individuals. The film captures the emotional toll of societal expectations and familial obligations on Mathew, who copes with his identity in an environment that offers little room for authenticity. Using the lens of gerontology, *“Kaathal: The core”* invites a deeper examination of how ageing intersects with queerness, particularly in spaces where heteronormative ideals dictate personal and social relationships. Gerontology, the study of ageing, offers a critical framework to understand how older queer individuals contend with societal pressures while negotiating their emotional, sexual and social lives.

#### 7.6.2 Narrative complexity and social critique

In the film, Mathew’s character arc is shaped by the tension between his public persona—a respected political candidate—and his private identity as a queer man. The internalized shame that Mathew carries is emblematic of the broader struggles faced by older gay men, who are often forced into silence due to societal norms. This silence is particularly pronounced in traditional family structures, where the concept of queerness is either ignored or vilified. The film addresses these tensions through nuanced dialogues and moments of introspection, highlighting the isolation and erasure that come with ageing as a queer individual. One of the most compelling aspects of *“Kaathal: The core”* is its critique of how older queer individuals are desexualized in popular media and public discourse. Sexuality, when portrayed in the context of older individuals, is often dismissed or treated as taboo, especially in conservative societies like India. For older gay men, this erasure is compounded by societal homophobia, which views queerness as an aberration that must be suppressed or corrected. Mathew’s relationship with Thankan, subtly implied through the film’s dialogues and character interactions, challenges this narrative by presenting queerness as a lifelong experience rather than a phase confined to youth. Their bond, while understated, is a powerful testament to the resilience of queer love in the face of societal stigma. However, the film also does not shy away from portraying the emotional toll this relationship takes on both men, particularly as they navigate a world that refuses to acknowledge their existence. The traditional family structure, as portrayed in the film, serves as both a site of comfort and conflict for Mathew. His marriage to Omana reflects the societal pressures that queer individuals face to conform to heteronormative ideals. Omana’s eventual confrontation of Mathew reveals the emotional cost of such arrangements, not only for the queer individual but also for their spouse. The dialogues between Mathew and Omana capture the pain of a relationship built on societal expectations rather than mutual understanding, shedding light on the collateral damage of compulsory heterosexuality. These moments of conflict are juxtaposed with Mathew’s interactions with his political colleagues, who represent the performative progressiveness of a society that claims to embrace diversity while perpetuating exclusionary practices. The film’s depiction of political hypocrisy underscores the challenges of navigating public life as a queer individual, particularly in spaces where personal identity becomes fodder for political agendas. In addition to its narrative depth, *“Kaathal: The core”* is significant for its contribution to the broader discourse on queer visibility in Indian cinema. By centering its story on an older gay man, the film disrupts the dominant narrative framework that prioritizes youth and romance in queer storytelling. It also raises important questions about the lack of institutional and community support for older queer individuals, who are often left to navigate their struggles in isolation. The film’s nuanced portrayal of Mathew’s character serves as a call to action for more inclusive and representative storytelling that acknowledges the diversity within the LGBTQ+ community.

#### 7.6.3 Intersectionality and broader implications

This study situates *“Kaathal: The core”* within the broader context of queer studies and gerontology, examining how the film addresses the intersections of age, queerness, and societal expectations. It argues for a more comprehensive understanding of queer ageing that goes beyond the limitations of existing representations, advocating for narratives that reflect the complexities of older queer individuals’ lives. By highlighting the emotional, sexual, and social dimensions of queer ageing, the film contributes to a growing body of work that seeks to challenge stereotypes and expand the scope of queer representation in Indian cinema. In doing so, *“Kaathal: The core”* not only amplifies the voices of older queer individuals but also invites audiences to rethink their assumptions about identity, love, and ageing. This study employs an intersectional approach to analyse the experiences of older gay men as portrayed in *Kaathal: The core*. The theory of Intersectionality, developed by Kimberlé Crenshaw, provides a nuanced lens for examining how the overlapping identities of age, gender, and sexuality shape the visibility and lived experiences of older LGBTQ+ individuals (Crenshaw, 1989). Originally conceptualized to highlight the compounded discrimination faced by Black women, Intersectionality Theory has since become a critical tool for understanding how multiple identities converge, producing unique forms of social disadvantage or privilege (Crenshaw, 1991). In the context of this study, an intersectional approach allows for a deeper exploration of how older gay men experience marginalization within a heteronormative, family-centric society that often enforces rigid gender and sexual norms. Applying intersectionality in this research enables an examination of how these men’s identities as both older and gay compound their challenges, particularly within the conservative social milieu of Kerala. As the protagonist confront social expectations tied to age and familial duty, as well as pressures stemming from societal rejection of queer identities, they navigate a complex landscape marked by both resilience and vulnerability. This approach underscores the necessity of analyzing older queer identities not in isolation but as products of intersecting forces such as, age, gender, sexual orientation, and cultural context, each influencing their portrayal and treatment in the film. The intersectional lens not only highlights the compounded stigma faced by older LGBTQ+ individuals but also provides insights into the ways these characters resist or conform to societal expectations, bringing visibility to the often-neglected dimensions of queer ageing in Indian cinema.

## 8 Implications

This research not only enriches the understanding of queer ageing and iconoclastic narratives in *Kaathal: The core* but also serves as a call to action on academics, filmmakers, and society at large. It advocates for more inclusive cinematic storytelling and cultural spaces that recognize the authenticity, dignity, and live experiences of LGBTQ+ individuals across age, class, and cultural boundaries.

### 8.1 Contribution to queer studies and intersectionality

By examining *Kaathal: The core*, this research expands the discourse on queer representation in Indian regional cinema, particularly focusing on the neglected theme of queer ageing. It highlights how heteronormative societal structures, and patriarchal expectations intersect with age and sexuality, leading to compounded marginalization. This study offers a deeper understanding of how intersectional identities—being queer, older, and part of a conservative society—are portrayed, providing a foundation for further exploration of underrepresented LGBTQ+ narratives.

### 8.2 Reshaping film criticism in regional cinemas

The research contributes to film criticism by emphasizing the role of regional cinema, particularly Malayalam cinema, in pushing the boundaries of queer storytelling. While much of mainstream Indian cinema remains hesitant to engage deeply with LGBTQ+ themes, *Kaathal: The core* serves as a landmark in depicting complex queer experiences. The study invites filmmakers and critics to recognize the importance of diverse, authentic narratives and to challenge stereotypes that persist in cinematic portrayals.

### 8.3 Impact on cultural conversations around queer ageing

The focus on queer ageing in *Kaathal: The core* calls attention to a topic that has remained largely invisible in cultural discourse. By portraying older gay men like Mathew and Thankan, the film confronts the emotional, social, and psychological realities of ageing queer individuals. This research encourages greater societal awareness of the challenges faced by older LGBTQ+ individuals and advocates for spaces of inclusion, empathy, and dignity for people at the intersections of age and sexual identity.

### 8.4 Challenging societal norms through iconoclasm

The film’s iconoclastic critique of heteronormative marriage, familial duty, and societal expectations opens critical reflections at the cost of silence and inauthenticity. This research emphasizes how cultural productions like *Kaathal: The core*, can function as a tool for resistance, challenging oppressive systems and advocating for more inclusive definitions of family, identity, and love. It provides a blueprint for rethinking societal norms that continue to marginalize queer individuals.

### 8.5 Academic and policy interventions

The findings from this research can inform academic studies on gender, sexuality, and cinema, providing a critical framework for analyzing LGBTQ+ narratives in regional film industries. Furthermore, it holders’ implications for policymakers and cultural advocates, urging them to foster environments that promote inclusive media representation and challenge systemic silences around queer identities.

## 9 Limitations

While this research provides a critical analysis of *Kaathal: The core* with a focus on queer ageing and iconoclastic narratives, certain limitations must be acknowledged. Firstly, the study is limited to a single film, while being significant, cannot fully encompass the breadth of queer representation and ageing experiences in Malayalam cinema or in Indian regional cinema at large. This singular focus restricts the generalizability of the findings, as other films may approach similar themes differently through narrative style, cultural contexts, or directorial intent. Secondly, the analysis relies heavily on narrative and visual elements, limiting the incorporation of external factors such as audience reception or societal impact, which are crucial in understanding how such portrayals influence cultural perceptions. The absence of empirical data—such as interviews with audiences, filmmakers, or members of the queer community—further narrows the study’s scope. A more holistic understanding of the film’s impact would require integrating primary data with textual analysis. Additionally, the research addresses the themes of queer ageing and societal silence, while it does not deeply examine how other intersecting factors—such as class, religion, and caste—affect the live experiences of the older queer individuals in Kerala. These intersections may play a pivotal role in shaping the characters’ struggle, which remains an area for further exploration.

As with any qualitative analysis, interpretations are influenced by the researcher’s cultural background, personal beliefs, and academic framework. While efforts were made to approach *Kaathal: The core* through established queer and gerontological theories, subjective interpretations may have shaped the analysis of character motivations, narrative themes, and cinematic techniques. The researcher’s cultural background, language familiarity, and personal experiences may have influenced the interpretation of themes in the film. To minimize bias, multiple viewings, secondary sources, and theoretical frameworks were employed for balance. Despite these efforts, some emotional or symbolic elements may invite alternative readings, underscoring the need for diverse scholarly perspectives on queer ageing in Indian cinema.

## 10 Future scope

This study opens avenues for further research on queer representation and ageing, in Indian regional cinemas. Future studies can expand the scope by analyzing a broader selection of films across different linguistic and cultural contexts within India, comparing how themes of queer ageing, societal pressure, and iconoclasm are depicted. A comparative analysis between Malayalam cinema and other Indian film industries, such as Bollywood, Kollywood, and Tollywood, can offer a deeper understanding of how regional cultural frameworks shape narratives in the case of queerness and ageing. Further research can also focus on audience reception to the films like *Kaathal: The core*. Examining how viewers interpret, accept, or challenge the film’s themes can provide insights into the cultural and social impact of such representations, bridging the gap between cinematic portrayals and real-life experiences. Empirical and Ethnographic studies involving interviews with older queer individuals, filmmakers, and LGBTQ+ activists can enrich the discourse, grounding the analysis in live experiences. Moreover, exploring how queer ageing intersects with other social identities—such as caste, class, and religion—can offer a more nuanced understanding of the challenges faced by older LGBTQ+ individuals in diverse socio-cultural contexts. This intersectional approach can shed light on the systemic barriers that remain invisible in single-axis analyses. To improve the representation of queer ageing, filmmakers should expand narratives beyond repression and tragedy, showcasing themes of resilience, empowerment, and fulfilling relationships. Further, cultural policymakers can support such portrayals by offering funding incentives, promoting inclusive narratives in public broadcasting, and encouraging representation in mainstream media. Authentic casting of older LGBTQ+ actors and consulting advocacy groups also can ensure accuracy and sensitivity. Future research can expand by incorporating audience reception studies to assess viewer interpretations, ethnographic research to gather firsthand experiences of older LGBTQ+ individuals, and longitudinal studies to track evolving queer representations in Indian cinema. Discourse analysis of media responses and comparative studies with international films can reveal cultural contrasts, while intersectional approaches can explore the role of caste, class, and religion in shaping queer ageing narratives. These methods would offer deeper insights into the complexities of queer identity and ageing in diverse contexts.

In concluding further scope, this research lays the foundation for understanding queer ageing and iconoclastic narratives in *Kaathal: The core*. Its limits open pathways for future research. A broader, intersectional, and empirical approach will further deepen our understanding of the complexities of queer lives and the role of cinema, in representing them authentically.

## 11 Conclusion

On 2 February 2016, the Supreme Court decided to review the criminalization of homosexual activity. On 6 September 2018, the Supreme Court unanimously struck down Section 377 as unconstitutional, ruling that it infringed on the fundamental rights of autonomy, intimacy, and identity, thus legalizing homosexuality in India, including in Kerala. This research underscores how *“Kaathal: The core”* serves as a powerful iconoclastic narrative that challenges the deeply entrenched societal, cultural, and institutional perceptions of identity, family, and ageing, within a heteronormative framework. By focusing mainly on queer ageing, the film disrupts conventional portrayals of LGBTQ+ lives in the Malayalam cinema, offering a rare and sensitive depiction of older gay men grappling with their identities amidst societal silence and repression. Characters, Mathew Devasy and Thankan represent individuals caught between personal authenticity and societal expectations, highlighting the cost of living truthfully in a culture that privileges conformity. The film embodies the burden of queer ageing, wherein the weight of unspoken truths forces individuals into the lives of emotional compromise. The film critiques not only heteronormativity but also patriarchal structures that entrap both LGBTQ+ individuals and their spouses. Together, the narratives challenge the institution of marriage as a tool for enforcing conformity while advocating for individual freedom and emotional authenticity.

By addressing queer ageing, a theme, largely neglected by Indian cinema, *“Kaathal: The core”* stands as a bolder iconoclastic text that dismantles societal assumptions about gender, sexuality, and age. It establishes a place within the larger trajectory of queer representation in Malayalam cinema, while marking a significant progression toward inclusive storytelling. This research highlights the importance of such narratives in fostering empathy and visibility, urging society to move beyond silence and shame, toward acceptance and celebration of diverse identities. In doing so, the film paves the way for a more equitable cultural landscape where the dignity of living authentically is not a privilege but a fundamental right. Queer aging, often invisible and marginalized in Indian society, is brought to the forefront in *Kaathal: The core*, challenging the status quo with its unapologetic portrayal of lived experiences. The film not only defies traditional notions of aging but also highlights the resilience and strength of older LGBTQ+ individuals. It serves as a mirror to the silenced voices of queer elders, who have long been sidelined in both cinematic and societal narratives. By showcasing their lives with dignity and grace, *Kaathal: The core* administers viewers to confront ageism alongside heteronormativity, reinforcing the idea that love, desire, and self-expression know no boundaries of time.

## Data Availability

The original contributions presented in the study are included in the article/supplementary material, further inquiries can be directed to the corresponding author.
